# Medical Society

**Published:** 1897-11-13

**Authors:** 


### MEDICAL SOCIETY.

At the last meeting, the President (Dr. A. E. Sansom) showed some cases of mitral stenosis, and, after describing the signs indicative of the various stages of the disease, insisted on the rheumatic origin of the majority of the cases occurring in youth. Dr. W. S. Colman showed a very interesting case in which a child without any mental defect—in fact, a very bright little specimen—had shown many of the usual characteristics of the cretinoid physique. Mr. C. Mansell Moullin exhibited two patients in whom tumours, which were presumed to be sarcomata, and which, from their position, were unsuitable for operation, had been treated by the injection of Coley's Fluid, and had almost disappeared. Several other cases were mentioned in which beneficial results had seemed to follow this treatment. On the other hand, Mr. Watson Cheyne, who had also used this fluid, while fully accepting the statements of Dr. Coley, was unable from his experience to speak favourably of the method.

